# Are allergen batch differences and the use of double skin prick test important?

**DOI:** 10.1186/s12890-015-0021-3

**Published:** 2015-04-09

**Authors:** Gert F Thomsen, Vivi Schlünssen, Lars R Skadhauge, Tine Halsen Malling, David L Sherson, Øyvind Omland, Torben Sigsgaard

**Affiliations:** Department of Occupational Medicine, Hospital of Southwest Denmark, Finsensgade 35, 6700 Esbjerg, Denmark; Department of Public Health, Danish Ramazzini Centre, University of Aarhus, Aarhus, Denmark; Department of Occupational Medicine, Danish Ramazzini centre, Aarhus University Hospital, Aarhus, Denmark; Institute of Regional Health Research, Faculty of Health Sciences, University of Southern Denmark, Odense, Denmark; Department of Occupational Medicine, Danish Ramazzini Centre, Aalborg University Hospital, Aalborg, Denmark; Department of Occupational Medicine, Odense University Hospital, Odense, Denmark; Department of Pulmonal Medicine, Odense University Hospital, Odense, Denmark; Department of Health Science and Technology, The Faculty of Medicine; Aalborg University Hospital, Aalborg, Denmark

**Keywords:** Skin prick test, Aeroallergens, Population based study, Validity, Rhinitis

## Abstract

**Background:**

Skin prick tests (SPT) are widely used both in clinical diagnostics and in research. The standardization of allergen extracts is well documented to be crucial for the validity of SPT, whereas less emphasis has been placed on reproducibility and the SPT procedure itself. The objectives of this study are to clarify how the double skin prick test procedure influence the sensitivity and specificity of the test and to analyse the differences in weal size in skin prick tests between two batches of allergen extracts from the same vendor.

**Methods:**

The association between rhinitis and SPT was assessed among 1135 persons from a general population sample. SPT was performed twice with 10 common aeroallergens. In a subsample of 90 persons SPT was performed simultaneously with five of the allergens using different batches.

**Results:**

Thirty percent had at least one positive SPT. Among asthmatics this number was 62%. Only minor differences were seen between the sizes of two weals from the same batch. A second SPT with the same batch did not change the association between rhinitis and sensitization. When performing SPT with two different batches disagreement was observed in 2% (Birch) to 11% (Cat) of the subjects.

**Conclusions:**

Performing SPT twice with the same allergen batch does not enhance the validity of the test, and value of double testing can be questioned. Considerable differences in SPT response with different batches from the same manufacturer were observed. Thus inter batch differences in allergen extracts might be a source of variability.

## Background

Both in daily clinical diagnostics and in epidemiological studies a valid and reproducible test for immediate type allergy is highly desirable. Skin Prick Test (SPT) has been widely used. The standardization of SPT allergens has been documented to be crucial for the validity of SPT [1-6], but there has been less emphasis on the reliability of the tests.

The tool used to apply the allergens have some impact on the reliability of the tests. Masse et al. compared four instruments in current use and showed, that the ALK lancet had high acceptability and the lowest variability [7].

According to the EAACI guidelines SPT should be performed in duplicate [8]. The value of doing this has been disputed. In children Devenney has shown, that the double SPT procedure only leads to differences in about 1% of the tests [9]. In a study comparing SPT allergens from different manufacturers van Kampen reports no significant differences in weal size between two pricks with the same allergen from the same source, but found large differences between allergens from different vendors [10], and these results were confirmed by others [3,6]. Less focus have been on variation between batches from the same manufacturer. In one study with patients referred to an allergy clinic Bjorksten showed significant variation in SPT reactions to different batches of allergen from the same vendor [11].

To our knowledge the variability between two batches from the same vendor applied simultaneously in a population-based study has never been performed. We had the opportunity to study this issue when some of the participants in the Danish RAV study (Risk factors for asthma in adults) [12] at the same time were enrolled in the GA2LEN Selenium project [13]. The objectives of the present study were to elucidate whether double SPT procedure enhances the sensitivity and/or specificity of the test and to analyse the differences in weal reactions in SPT’s with two different batches of allergen extracts from same vendor.

## Methods

### Study population

This study is part of the RAV study; a Western Denmark five centre study on risk factors for asthma in adults [12]. From the catchment area of each of the five centres 2,000 subjects aged 20–44 standardized by gender and age were randomly selected from the Danish Civil Registration System. In 2001–2003 an extended version of the ECRHS II screening questionnaire (www.ECRHS.org) was sent to these 10,000 persons. 7271 responded. Among responders a random sample of 20 percent and a symptom group comprising all subjects who reported currently taking any medicine for asthma, asthma attack, or woken by an attack of shortness of breath at any time in the past 12 months were invited to a clinical investigation in 2003–2006.

The clinical investigation consisted of an interviewer-administered questionnaire based on the ECRHS II protocol [14], SPT, blood samples, spirometry and measurement of non-specific bronchial responsiveness.

Asthma, Rhinitis and smoking status was obtained from the interview. Current asthma was defined as an affirmative answer to the question “Are you currently taking any medication (including inhalators, aerosols or tablets) for asthma?” or the combination of an affirmative answer to the question “ Has your asthma ever been confirmed by a doctor?” and at least one of the following questions “Have you had wheezy breathing at any time during the last 12 months?”, “Have you woken up with a feeling of chest tightness at any time during the last 12 months?”, “Have you had breathlessness at rest in the daytime during the last 12 months” or “Have you woken up with an attack of breathlessness at any time during the last 12 months?”. “Current rhinitis” was defined as an affirmative answer to the question “Have you had problems of sneezing, running or stuffy nose without having a cold or flu within the last 12 months”. The subject was then asked in which months he/she had these problems. Seasons of the allergens were defined based on data from Danish Meteorological Institute 2003 [15]. Birch: April-May, Grass: June-July, Mugwort: July-August, *Alternaria*: July-September and *Cladosporium*: June-September. Seasonal rhinitis was defined when symptoms in all the months of the given allergen season was reported. Subjects with perennial symptoms were not included in the seasonal rhinitis group.

Dust mites, cat, dog and horse allergen were classified as “Perennial allergens”. Perennial rhinitis was diagnosed if the participant mentioned symptoms in at least 8 months of the year or could not specify “symptom months”.

### Skin prick test

The participants were asked to refrain from any antihistaminic medication 72 hours before the visit. SPT was carried out with commercial allergens from Alk-Abelló: Pollens: Birch, Grass (*Phleum pratense*) and Mugwort (*Artemissa Vulgaris*). Danders: Cat, Dog and Horse. Moulds *Alternaria* (*Alternaria Alternata*) and *Cladosporium* (*Cladosporium Herbarum*). House dust mites *Dermatophagoides Pteronyssinus* (*D pter*) and *Dermatophagoides Farinae* (*D. Farinae)*. Positive control: Histamine 10 mg/ml. Negative control: 50% Glycerol in aqua solution. After disinfection with alcohol solution ALK number tape was placed on the volar side of the forearm and droplets of allergen extract were applied at one side of the tape in a predefined order. For each allergen a Phazet lancet was pricked perpendicular through the droplet. Double SPT: With the same lancet a second prick was made at the opposite side of the tape, using the allergen extract on the lancet from the first prick. This procedure was chosen above placing another drop of allergen to reduce the necessary time as well as allergen spending. After 15 minutes positive, negative and all allergen reactions were drawn up on the skin and transferred with transparent adhesive tape onto a record sheet. The SPTs were carried out by either physicians or especially trained nurses. The largest diameter and the perpendicular diameter were measured by two readers. If different, further measurements were made until agreement between the readers was obtained. (Largest diameter + perpendicular diameter)/2 > = 3 mm was considered as positive reaction and the actual weal size was recorded. If < 3 mm the result was recorded as negative as is usual in clinical settings.

In one centre a subsample of participants were at the same time enrolled in the GA2LEN Selenium project [13], namely 55 subjects from the random sample of the RAV study and 36 subjects from the RAV symptomatic sample who also fulfilled the GA2LEN asthma control and case criteria. Cases were subjects with a self-reported diagnosis of asthma and either wheezing, shortness of breath or waking at night with breathlessness in the previous 12 months. Controls were subjects with neither a diagnosis of asthma nor any of the three symptoms. In these subjects SPT’s were performed simultaneously with 10 allergens on the right forearm (The RAV Study) and 7 allergens on left forearm (The GA2LEN Study). All Allergens were delivered from ALK Abello. 5 allergens were in common (Birch, Grass, Cat, *D Farinae* and *Alternaria*), but from different production batches. The declared allergen activity (Birch, Grass, Cat, *D Farinae*) or concentration (*Alternaria*) did not vary between batches. The GA2LEN allergens were only pricked once. In this subgroup, the actual weal size of all skin pricks were recorded, even when less than 3 mm. In the analysis of the correlation between SPT with different batches, the first of the two RAV study pricks was used.

The term disagreement between two SPT’s is used, when one is positive and the other negative.

### Statistics

Agreements in positive tests for each allergen were calculated using Kappa Statistics. To compare the ability of the tests to identify subjects with rhinitis the sensitivity and the specificity were calculated. When choosing the cut-off value for a test there will always be a trade-off between sensitivity and specificity. To encompass both, the Youden’s indexes [16] were calculated. The Pearson correlation coefficients were calculated to describe the correlation between the weal sizes of the two skin pricks in the “two batches” procedure. The difference between intrabatch variability and interbatch variability were tested as follow: For each participant the difference between the sizes of the two weals with same batch (Intrabatch) as well as the difference between Prick1 batch1 and prick batch 2 (Interbatch) were calculated. The equality of the interbatch and intrabatch differences were tested by Student’s t-test. The differences in number of disagreements were tested by Wilcoxon signed-rank test. As five comparisons are performed, p < = 0.01 was chosen for reporting significant results. All statistical analyses were conducted in Stata 10.0 IC (Stata Cooporation Texas, USA).

### Ethical considerations

The study was approved by the Medical Ethics Committee of Ribe & Sonderjyllands County, Fyn & Vejle County, and Nordjyllands County. All subjects signed an informed consent form before participating in the study.

## Results

1.191 subjects attended the clinical examinations. Thirty-four subjects refused SPT and furthermore 22 subjects refused the double SPT procedure leaving 1,135 subjects eligible for analysis. Due to shortage of allergen extracts SPT for *Cladosporium* was not performed in 114 subjects and for *Alternaria* in 59 subjects. Among the 91 subjects who participated in both the RAV and the GA2LEN study one subject refused SPT leaving 90 subjects eligible for analysis. Among these, 69 subjects accepted both double SPT with the RAV allergens and SPT with the GA2LEN allergens.

The average age of the population was 34 years, and 55% was females. In the random sample the prevalence of current asthma was nine percent and current rhinitis 43%, Table 1.

**Table 1:** **Number of participants, demographics and frequency of asthma and rhinitis**

	**Random sample**	**Symptom sample**	**Total**
Total RAV population N	708	427	1135
GA2LEN + RAV SPT N	54	36	90
RAV SPT only N	654	391	1044
Age (SD)	33.7 (7.0)	33.7 (7.1)	33.7 (7.1)
Female (%)	379 (53.5)	245 (57.4)	624 (55.0)
Asthma (%)	66 (9.3)	211 (49.4)	277 (24.4)
Rhinitis (%)	303 (42.8)	288 (67.5)	591 (52.1)

The prevalence of positive SPT in the total RAV cohort is presented in Table 2. In the random sample of the RAV study the prevalence varied from 1% for *Cladosporium* to 18% for grass. The prevalence of SPT positive reaction to at least one allergen was 31%, in asthmatics 62%. The prevalence of SPT in the symptom sample was, as expected, increased compared to the random sample. In the random sample the prevalence was higher among < 25 years compared to ≥ 25 years), 39% vs. 29% (p < 0.05), whereas for the symptom sample the prevalence was similar across age-groups, ≈ 50%.

**Table 2:** **Results of SPT’s in all centres**

		**SPT Batch 1**		**Random Sample N = 708**	**Symptom Sample N = 427**	**Current Asthma N =277**
		**Prick 1**	**Prick 2**			
**Allergen**	**N**	**Pos (%)**	**Pos (%)**	**% pos**	**% pos**	**% pos**
*Cladosporium*	1021	14 (1.4)	17 (1.7)	1.1	1.9	3.9
*Alternaria*	1076	45 (4.2)	46 (4.3)	1.9	8.5	12.6
Mugwort	1135	70 (6.2)	70 (6.2)	4.5	8.9	10.5
Grass pollen	1135	268 (23.6)	270 (23.8)	17.7	33.5	39.0
Birch pollen	1135	171 (15.1)	164 (14.4)	11.6	20.8	22.7
*D. Farinae*	1135	170 (15.0)	175 (15.4)	10.0	23.2	31.1
*D. Pter.*	1135	208 (18.3)	206 (18.1)	12.7	27.6	37.2
Cat	1135	143 (12.6)	149 (13.1)	7.6	20.8	27.4
Dog	1135	151 (13.3)	159 (14.0)	7.1	23.7	30.3
Horse	1135	56 (4.9)	48 (4.2)	2.8	8.4	11.6
Any allergen	1135	442 (38.9)	447 (39.3)	30.5	51.1	62.1

**Table 3:** **SPT double prick using same batch of allergen extract**

	**All: 1135**		**Asthmatics: 277**	
**Allergen**	**Kappa**	**Dis-agreements**	**Kappa**	**Dis-agreements**
*Cladosporium*	0.90	0.3%	0.90	0.8%
*Alternaria*	0.83	1.4%	0.81	4.5%
Mugwort	0.87	1.4%	0.86	2.5%
Grass	0.93	2.5%	0.92	3.6%
Birch	0.91	2.2%	0.92	2.9%
*D. Farinae*	0.92	2.0%	0.91	4.0%
*D. Pter.*	0.92	2.5%	0.92	4.0%
Cat	0.94	1.3%	0.93	2.9%
Dog	0.82	4.0%	0.84	6.9%
Horse	0.90	0.8%	0.89	2.2%

Positive result in prick 1 vs prick 2.

### Double SPT procedure

A high degree of concordance was seen between the two weals when SPT was carried out with the same allergen extract. The correlation coefficients varied from 0.88 in *Alternaria* and dog to 0.94 for horse and birch. The reactions to Dog allergen showed the highest rate of disagreements: 4%. A higher percentage disagreements was seen in the asthma group, whereas the Kappa values were the same, Table 3. Disagreements were seen in both directions except for *Cladosporium*. In 87% of the 1,135 participants, agreement was seen for all 10 allergens.

### Differences between batches

For Grass and Cat allergens the SPT mean weal size was significantly greater for the GA2LEN panel than the RAV panel: Grass 3.1 vs. 2.1 mm and cat 2.1 vs. 1.1 mm. For *Alternaria*, birch and *D Farinae* no significant differences in mean weal size was seen. The Pearson correlation coefficients between the average diameters in the two tests varied from 0.79 for grass to 0.94 for *D Farinae*. The percent disagreement in the 90 subjects varied from 2.2 for birch to 11.1 for cat, Table 4.

**Table 4:** **SPT using two different batches of allergen extracts**

**N = 90**	**SPT Batch 1 RAV**	**SPT Batch 2 GA2LEN**			
**Allergen**	**Pos (%)**	**Pos (%)**	**Pearson ρ for wealsize**	**Kappa for positive test**	**Disagreements%**
*Alternaria*	8 (8.9)	10 (11.1)	0.91	0.75	4.5
Grass	29 (32.2)	35 (38.9)	0.80	0.81	8.9
Birch	16 (17.8)	18 (20.0)	0.96	0.93	2.2
*D. Farinae*	21 (23.3)	22 (24.4)	0.94	0.91	3.3
Cat	19 (21.1)	25 (27.8)	0.89	0.70	11.1

### Inter batch and intra batch variation

For five allergens it was possible to compare the intra- and inter batch variation in 69 subjects. In 54 participants (78%) agreement between batches was seen for all five allergens. The rate of disagreement was higher and the Kappa value lower for two different batches compared to double SPT with the same batch. This was true for all 5 allergens. For *Alternaria*, Grass and Cat the between batch differences in weal sizes were significantly larger than the within batch differences. When comparing number of disagreements significance was seen for Cat only Table 5.

**Table 5:** **Repeated SPT with different and with same batch**

**N = 69**	**Different batches**				**Same batch**			
**Allergen**	**Pearson ρ for weal size**	**Kappa for positive**	**Dis-agreements**	**Mean difference mm**	**Pearson ρ for weal size**	**Kappa for positive**	**Dis-agreements**	**Mean difference mm**
*Alternaria*	0.84	0.68	5.8%	0.9	0.92	0.92	1.5%	0.3*
Grass	0.79	0.76	11.6%	1.2	0.92	0.90	4.4%	0.6*
Birch	0.97	0.95	1.4%	0.4	0.94	1.00	0%	0.3
*D farinae*	0.94	0.90	4.3%	0.6	0.91	0.93	2.9%	0.5
Cat	0.91	0.75	10.1%	1.2	0.92	1.00	0% ¤	0.4*

¤p < 0.01 in Wilcoxon signed-rank test *p < = 0.005 in t-test.

### Allergy tests and rhinitis

Sensitivity, specificity and Youdens index for any positive SPT were calculated using rhinitis as the “true diagnosis” (Sensitivity = 0.57, specificity = 0,76 and Youdens index = 0.33). The same calculations were made for positive reaction to any perennial allergen with perennial rhinitis as the “true diagnosis” (0.37, 0.74 and 0.11) and finally for seasonal rhinitis with symptoms in the birch, grass and mugworth pollen seasons as the “true diagnosis”. For the single allergens the sensitivity were lower and the specificity higher (Table 6) Subgroup analysis on the random group and the asthma symptom group showed similar results (data not shown).

**Table 6:** **The ability of double SPT compared to single SPT to identify persons reporting rhinitis last 12 months**

**All N = 1135**	**SPT result**	**Positive reactions**	**Sensitivity**	**Specificity**	**Youden index (SE)**
Rhinitis any time during the year	Any allergen positive	Prick 1	0.56	0.77	0.33 (0.03)
		Prick 1 or 2	0.58	0.75	0.33 (0.03)
		Prick 1 and 2	0.55	0.79	0.34 (0.03)
Perennial rhinitis	Any whole year allergen positive	Prick 1	0.37	0.74	0.11 (0.04)
		Prick 1 or 2	0.39	0.73	0.12 (0.04)
		Prick 1 and 2	0.36	0.77	0.12 (0.04)
Birch season rhinitis	Birch positive	Prick 1	0.23	0.87	0.11 (0.03)
		Prick 1 or 2	0.24	0.86	0.11 (0.03)
		Prick 1 and 2	0.22	0.88	0.11 (0.03)
Grass season rhinitis	Grass positive	Prick 1	0.47	0.86	0.32 (0.03)
		Prick 1 or 2	0.48	0.84	0.33 (0.03)
		Prick 1 and 2	0.45	0.86	0.32 (0.03)
Mugworth season rhinitis	Mugworth positive	Prick 1	0.11	0.95	0.06 (0.02)
		Prick 1 or 2	0.12	0.95	0.07 (0.02)
		Prick 1 and 2	0.10	0.96	0.07 (0.02)

To analyse whether double SPT improved the diagnostic capability of the test, Prick 1 pos. was compared to (Prick 1 pos. OR Prick 2 pos.) and to (Prick 1 pos. AND Prick 2 pos.). As expected using (1 OR 2) showed a slightly higher sensitivity and lower specificity, while using (1 AND 2) lead to the reverse result. The Youden’s index’s hardly changed, Table 6. In the current asthma group the same patterns were seen, Table 7.

**Table 7:** **The ability of double SPT compared to single SPT to identify persons reporting rhinitis last 12 months**

**Current asthma N = 277**	**SPT result**	**Positive reactions**	**Sensitivity**	**Specificity**	**Youden index (SE)**
Rhinitis any time during the year	Any allergen positive	Prick 1	0.71	0.58	0.29 (0.06)
		Prick 1 or 2	0.73	0.57	0.30 (0.06)
		Prick 1 and 2	0.70	0.63	0.33 (0.06)
Perennial rhinitis	Any whole year allergen positive	Prick 1	0.60	0.50	0.09 (0.07)
		Prick 1 or 2	0.61	0.47	0.09 (0.07)
		Prick 1 and 2	0.58	0.52	0.10 (0.07)
Birch season rhinitis	Birch positive	Prick 1	0.21	0.76	0.03 (0.05)
		Prick 1 or 2	0.22	0.75	0.03 (0.05)
		Prick 1 and 2	0.20	0.79	0.02 (0.05)
Grass season rhinitis	Grass positive	Prick 1	0.53	0.73	0.25 (0.06)
		Prick 1 or 2	0.50	0.73	0.26 (0.06)
		Prick 1 and 2	0.50	0.73	0.23 (0.06)
Mugworth season rhinitis	Mugworth positive	Prick 1	0.13	0.92	0.06 (0.04)
		Prick 1 or 2	0.15	0.92	0.07 (0.04)
		Prick 1 and 2	0.13	0.94	0.07 (0.04)

These calculations were not performed for moulds due to few cases.

## Discussion

In this population based study we found a substantial number of disagreements in SPT using two different batches of the same allergen extracts from the same vendor. Performing double SPT using the same batch of allergen extract did not enhance the SPT validity. This was true for both the total group and for the asthma group.

Even though rather strict criteria were used (Doctors diagnose or current asthma medication) the prevalence of asthma in the random sample (9.3%) was higher than seen in population based questionnaires, around five percent [17]. This indicates that selection bias might be in play, when the participants choose to take part in the clinical investigations. As intra-individual differences are the focus in the analysis, we do not expect this selection to bias the results.

The SPT’s were performed following international guidelines. Standardized extracts from a well established vendor were used. All the of the weal size readings were double checked. The SPT’s were performed at five centres, but following the same protocol. At least one researcher from each centre participated in a common training session supervised by an acknowledged allergologist. In the same batch double SPT procedure, only one drop of allergen extract was used for both pricks. As the differences between weals were small and in both directions, it is not likely, that this led to significant bias.

The number participants in the between batch part of the study was determined by the number included in the Ga2len study. As very little has been published on differences in prick test reaction between bathes of the same allergen, we did not a priory have sufficient data to make a proper power calculation.

A total of 30.5% of the randomly selected subjects had at least one positive SPT. This is slightly less than the 33.9% reported by Linneberg in a Danish population based study in 1998 [18]. This difference might be explained by a different age distribution, as the subjects in Linnebergs study were younger (15–22) and the prevalence of sensitization is generally higher among younger adults [17]. The very low discrepancy in the double SPT procedure is in line with van Kampen’s findings in occupational allergens [10]. The current study gives no clue which way to interpret a double SPT with discordant results, as the Youden indexes barely vary between the “or” and the “and” interpretation with no clear direction. In current recommendations double SPT is recommended, but this is presumably based on general consideration about precision and not based on a systematic data analysis [19]. Furthermore, the between batch variability is considerably larger than the within batch variability. Thus nothing is gained by performing double prick tests with the same batch.

We use rhinitis as the “true diagnosis” when evaluating the validity of the SPTs. More often SPT is used to validate questionnaires or interviews (e.g. Karakaya [20] and Smith [21]). Asthma could have been used as the target, as both interview and spirometry data were collected for all subjects. Asthma though, is less closely related to allergy/atopy. Even in this younger population a substantial fraction of asthma cases are non-allergic. Furthermore rhinitis symptoms are more closely related to atopy (Braun-Fahrlander [22]) and used as a proxy for atopy in some studies (e.g. Schlunssen [23]).

Using the reported months with rhinitis symptom as an indicator for the causative allergen has limitations. Except for birch pollen the seasons of the allergens are overlapping, and other pollens may play a role eg. hazelnut in the spring time. The interviews were performed all year round and many participants had difficulties pointing out specific months with symptoms. Since the SPT’s were performed after the interview any bias from these uncertainties is expected to be non-differential, but might partly explain the low observed sensitivities.

The differences in SPT reactions between two batches from the same vendor are less than reported by Nielsen [6] comparing allergen extracts from different vendors. In some allergens (e.g. birch) the number of positive reactions did not differ much, whereas in others (e.g. Cat) larger differences was seen. Because the differences for nearly all allergens were seen in both directions and varied considerably between the allergens it is unlikely, that the differences could be explained by study design issues.

In all allergens except *D. Farinae* a higher rate of discrepancies and lower correlations were observed when comparing two different batches than when comparing two SPT with the same batch. This underlines the necessity to use the same batches of prick test allergens in multicentre studies. When SPT is repeated over a longer period it might not be possible to use the same batches over time. In these cases interbatch differences should be considered a possible source of variability.

The evaluation of variation between allergen batches was performed with only 5 allergens in just 90 subjects and only one vendor. The results should therefore be interpreted with some caution. The study should be repeated with at least all allergens in the recommended standard panel and on a larger number of subjects. It might also be of interest to test whether similar results are found in allergens from other manufacturers.

## Conclusions

Performing SPT twice with the same allergen batch does not enhance the validity of the test, and double testing cannot be recommended. Considerable differences in SPT responses with different batches from the same vendor were observed. Thus inter batch differences in allergen extracts might be a source of variability when SPT reactions are followed over time or compared between centres.

## Abbreviations

*D. Farinae*: *Dermatophagoides Farinae*
*D. Pter*: *Dermatophagoides Pteronyssinus*
SPT: Skin prick test
